# Detection of microstructural white matter alterations in functional gastrointestinal disorders assessed by diffusion kurtosis imaging

**DOI:** 10.1002/jgh3.12375

**Published:** 2020-06-12

**Authors:** Toshimi Chiba, Kenji Ito, Futoshi Mori, Makoto Sasaki, Takayuki Matsumoto

**Affiliations:** ^1^ Division of Internal Medicine, Department of Oral Medicine Iwate Medical University Morioka Japan; ^2^ Division of Ultrahigh Field MRI, Institute for Biomedical Sciences Iwate Medical University Yahaba Japan; ^3^ Division of Gastroenterology, Department of Internal Medicine Iwate Medical University Morioka Japan

**Keywords:** diffusion kurtosis imaging, functional gastrointestinal disorders, irritable bowel syndrome, functional dyspepsia

## Abstract

**Background and Aim:**

We evaluated whether diffusion kurtosis and tensor imaging (DKI/DTI) could reveal microstructural alterations in the brains of patients with functional gastrointestinal disorders (FGIDs), and whether imaging findings were correlated with health‐related quality of life (HRQOL).

**Methods:**

Twelve patients with FGIDs fulfilling the Rome IV criteria, and seven healthy controls were examined using a 3 T magnetic resonance (MR) scanner. Tract‐based spatial statistics and regions of interest analyses were performed to compare the mean kurtosis (MK), fractional anisotropy (FA), and mean diffusivity (MD) between patients with FGIDs versus controls. HRQOL was assessed in patients with FGIDs using the eight‐item short form of the Medical Outcome Study Questionnaire (SF‐8) and the Gastrointestinal Symptom Rating Scale.

**Results:**

Patients with FGIDs had extensive, widespread regions of reduced MD in the white matter in comparison with healthy controls, whereas no significant differences were observed in MK and FA. No significant differences in deep gray matter for the MK, FA, and MD values were observed between patients with FGIDs and controls. In patients with FGIDs, the FA values in the globus pallidus had a significant and negative correlation with SF‐8 (a mental component summary) (*r* = −0.797, *P* = 0.01 uncorrected for multiple comparisons).

**Conclusions:**

DKI/DTI can help identify microstructural white matter alterations in patients with FGIDs. The FA values in the globus pallidus may be useful for a severity assessment of FGIDs.

## Graphical abstract

We evaluated whether DKI/DTI could reveal microstructural alterations in the brains of patients withFGIDs, and if imaging findings were correlated with HRQOL. Patients with FGIDs had extensive, widespread regions of reduced MD in the white matter in comparison with healthy controls. In FGIDs, the FA values in the globus pallidus had a significant and negative correlation with SF‐8 (a mental component summary). The FA values in the globus pallidus may be useful for a severity assessment of FGIDs.

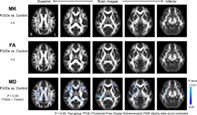

## Background

Functional gastrointestinal disorders (FGIDs) are characterized by visceral pain and discomfort. However, FGID pathophysiology has not been well characterized. FGIDs include irritable bowel syndrome (IBS) and functional dyspepsia (FD), both diagnosed using the Rome IV criteria. Alterations in brain–gut interactions with FGIDs could be related to the cause of the abdominal symptoms. Neuroanatomical differences among FGIDs have been investigated to clarify the symptomatology.^1^

Diffusion tensor imaging (DTI) is an advanced magnetic resonance (MR) technique widely used to quantify the diffusivity and/or anisotropy of water diffusion in the brain. However, few studies have studied white matter alterations in patients with IBS or FD compared to healthy controls. Previous DTI studies revealed changes in fractional anisotropy (FA) and/or mean diffusivity (MD) of specific white matter (WM) and deep gray matter (GM) structures in patients with IBS or FD.^2^, ^3^ Another advanced technique, diffusion kurtosis imaging (DKI), can reveal minute histological changes of complex brain structures via quantification of the degree of non‐Gaussian water diffusion.^4^ DKI has been reported to elucidate changes in periaqueductal GM (PAG) mean kurtosis (MK) and MD values among patients with migraine, suggesting that PAG changes may be involved in the underlying pathophysiology of migraine.^5^ However, it remains unknown whether DKI can detect subtle changes in brain tissue with FGIDs.

Therefore, the present study evaluated whether DKI/DTI can reveal microstructural alterations in brain with FGIDs and whether these imaging findings correlate with health‐related quality of life (HRQOL).

## Methods

### *Subjects*

We prospectively recruited 12 consecutive patients with FGIDs who visited our hospital for clinical assessment between December 2015 and August 2018. Subjects included eight patients with IBS (six with diarrhea‐predominant IBS and two with constipation‐predominant IBS) and four patients with FD. The clinical diagnoses of IBS and FD were established by a board‐certified gastroenterologist (TC) in accordance with the criteria of the Rome IV. The evaluation included upper or lower gastrointestinal endoscopy without organic abnormalities, hepatic function, renal function, and routine analysis of the blood. Exclusion criteria included mental deficiency, history of head trauma with loss of consciousness, drug abuse, neurological or psychiatric disorders, renal, hepatic, respiratory or cardiovascular illnesses, pregnant or lactating women, and contraindication to an MRI scan. In addition, seven healthy controls without any neuropsychiatric disorders, a history of acute or chronic disease, and any gastrointestinal symptoms were included as controls. The study was approved by the Human Ethics Review Committee of Iwate Medical University, and written informed consent was obtained from each subject prior to enrollment.

## Tesla MR imaging

### *Imaging protocol*

All subjects underwent MR examination using a 3T scanner (Trillium Oval, Hitachi, Ltd., Tokyo, Japan) with an eight‐channel head coil. DKI/DTI source images were acquired using a single‐shot spin‐echo echo‐planar imaging (EPI) method, with the following scan parameters, which were optimized^6^ and used in previous studies^5^, ^7^, ^8^, ^9^, ^10^: repetition time/echo time, 4500/110 ms; motion‐probing gradients, 20 directions; *b*‐values, 0, 1000, and 2500 s/mm^2^; field of view, 24 cm; matrix size, 128 × 128; reconstructed matrix size, 256 × 256; slice thickness, 3.0 mm without interslice gaps; number of slices, 36; number of excitations, 4; reduction factor of parallel imaging, 2; and acquisition time, 12 min, 18 s. Structural images, including three‐dimensional T1‐weighted, T2‐weighted, and fluid‐attenuated inversion recovery images, were also acquired.

### *Image analyses*

One of the authors (KI), who was blinded to information about subjects, conducted the DKI/DTI processing. Diffusion metric maps, including MK, FA, and MD, were calculated using an in‐house software program^11^ that has been used in previous studies.^5^, ^7^, ^8^, ^9^, ^10^ In the present study, FA and MD values were calculated using DTI data only from *b*‐values of 0 and 1000 s/mm^2^.

To identify changes in the WM of FGIDs patients, voxelwise statistical analyses of the MK, FA, and MD maps were conducted for screening the whole brain using tract‐based spatial statistics (TBSS) implemented on an FSL 5.0.9 instrument (FMRIB, Oxford).^12^, ^13^ Following skull stripping, FA maps were aligned into FMRIB58‐FA standard space using FMRIB's Non‐linear Image Registration Tool. The mean FA image of each subject was created, thinned, and set at a FA threshold of >0.20 to generate a mean FA skeleton representing the centers of the major WM tracts common to all subjects. The mean FA skeleton was masked to extract only the cerebral WM using the Harvard‐Oxford Subcortical Structural Atlas implemented in FSL, which has been used previously.^10^, ^14^ The FA map for each subject was then projected onto this skeleton to yield skeletonized FA maps. The MK and MD maps were also projected onto the mean FA skeleton by applying the same transformation as used for the FA map.

A voxelwise comparison of the MK, FA, and MD values was conducted for patients with FGIDs versus healthy controls with age and gender as covariates. The threshold for statistical significance was *P* < 0.05, using threshold‐free cluster enhancement (TFCE) with familywise effort (FWE) correction for multiple comparisons (corrected *P* < 0.05, 5000 permutations).

To perform deep GM ROI analyses, the Johns Hopkins University (JHU) Eve atlas was warped to the native space of each subject using inverse transformation matrices obtained by registration of the FA and b0 maps into the corresponding maps of the JHU‐Eve atlas via methods similar to those described previously.^8^, ^9^, ^10^ The ROI associated with the PAG, which was manually segmented on the averaged images of healthy controls,^2^ was also applied to the JHU‐Eve atlas. The mean MK, FA, and MD values of the caudate nucleus (CN), putamen (PU), globus pallidus (GP), thalamus (TH), substantia nigra (SN), red nucleus (RN), and PAG were then automatically measured using the coregistered ROIs.

### *Health‐related quality of life*

We measured the general and disease‐specific HRQOL using two types of instruments. The general HRQOL was assessed with the eight‐item short form of the Medical Outcome Study Questionnaire (SF‐8), and the disease‐specific HRQOL was assessed with the Gastrointestinal Symptom Rating Scale (GSRS) in FGIDs patients on the day of the MRI. SF‐8 and GSRS can be used to assess QOL for both upper and lower gastrointestinal diseases in FGIDs using the same parameters.

#### *SF‐8*

The SF‐8 is an HRQOL assessment consisting of eight scales. The summary measures generated are the physical component summary (PCS) and the mental component summary (MCS).^15^

#### *Gastrointestinal Symptom Rating Scale*

The GSRS is a disease‐specific instrument of 15 items combined into five symptom clusters describing reflux, abdominal pain, indigestion, diarrhea, and constipation. The reliability and validity of the GSRS is well documented.^16^

### *Statistical analysis*

The Mann–Whitney U test or Fisher's test was used to compare the median values of age or distribution of gender as well as to compare the median MK, FA, and MD values obtained by the ROI analysis between the patients with FGIDs and healthy controls. The correlation between the proportions of DKI/DTI and SF‐8 or GSRS was evaluated using Pearson's correlation test. In addition, the false‐discovery‐rate (FDR) correction was also used to correct for multiple comparisons. A *P* value <0.05 was considered statistically significant.

## Results

All the subjects underwent MR imaging and were eligible for further analyses. There were no significant differences in age or gender between patients with FGIDs and healthy controls (Table 1). No pathological findings, including asymptomatic infarction, were observed on structural imaging for any patient.

**Table 1:** Demographics of patients with functional gastrointestinal disorders (FGIDs) and healthy controls

	FGIDs (*n* = 12)	Controls (*n* = 7)	*P* value*
Age (years) (median)	31–67 (47)	25–79 (31)	0.14
Gender (male)	7 (58%)	6 (86%)	0.33
SF‐8 (MCS)	33.4–54.9 (48.0)	—	—
GSRS (abdominal pain score)	1.00–3.33 (1.67)	—	—

* Mann–Whitney *U* test or Fisher's test.

Data are presented as range (median) or *n* (%).

GSRS, the Gastrointestinal Symptom Rating Scale; MCS, the mental component summary; SF‐8, the eight‐item short form of the Medical Outcome Study Questionnaire.

**Figure 1:**
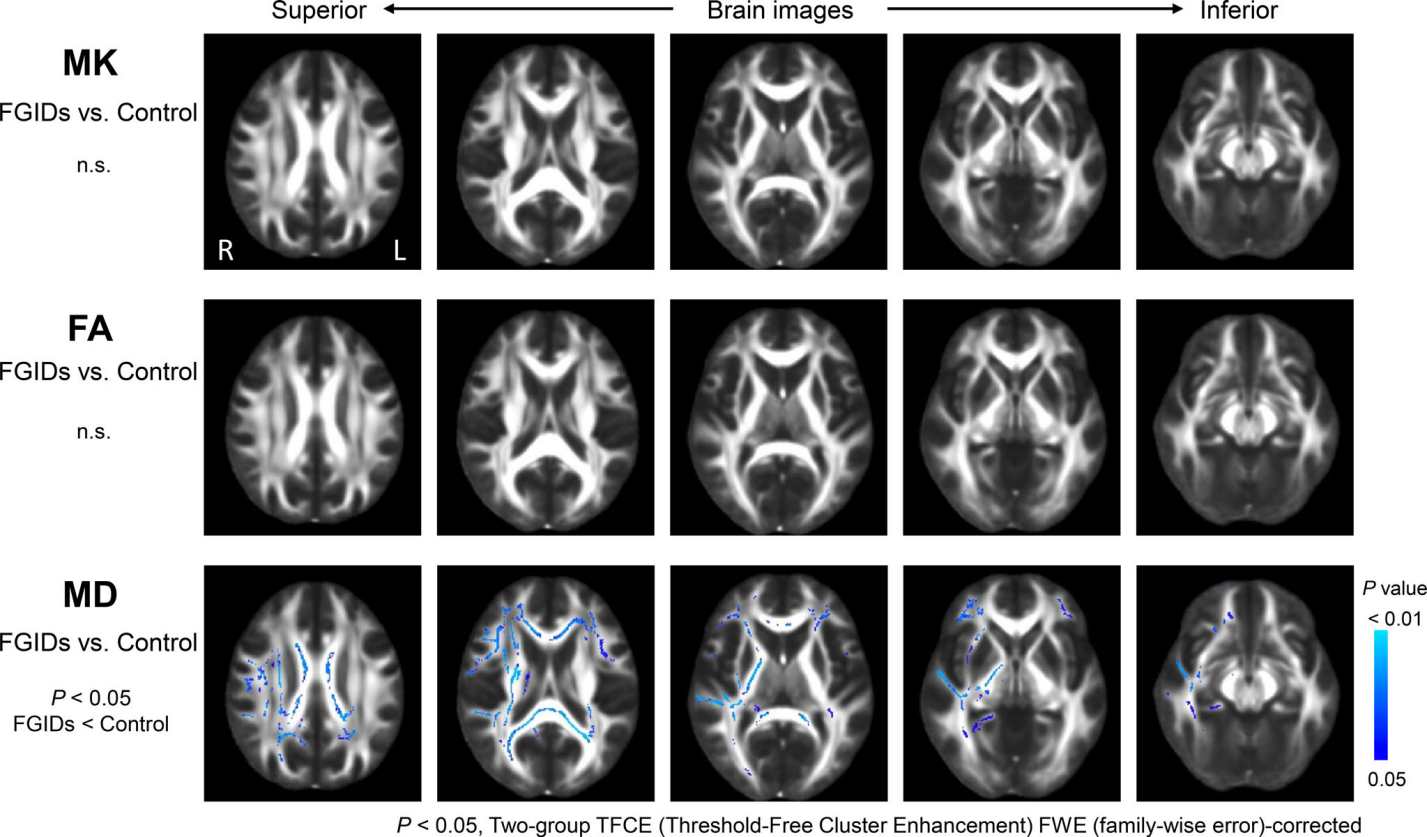
Voxelwise statistical analyses of the diffusion kurtosis and tensor maps of the white matter skeleton using tract‐based spatial statistics. Compared with healthy controls, patients with FGIDs showed significantly decreased mean diffusivity (MD) in widespread regions. Interestingly, no significant differences were observed in mean kurtosis (MK) and fractional anisotropy (FA) between the two groups. The statistical maps are overlaid on the mean FA map. Clusters that survived FWE correction of *P* < 0.05 with TFCE are presented as a colored area showing significant changes in the white matter skeleton. (n.s., not significant).

**Figure 2:**
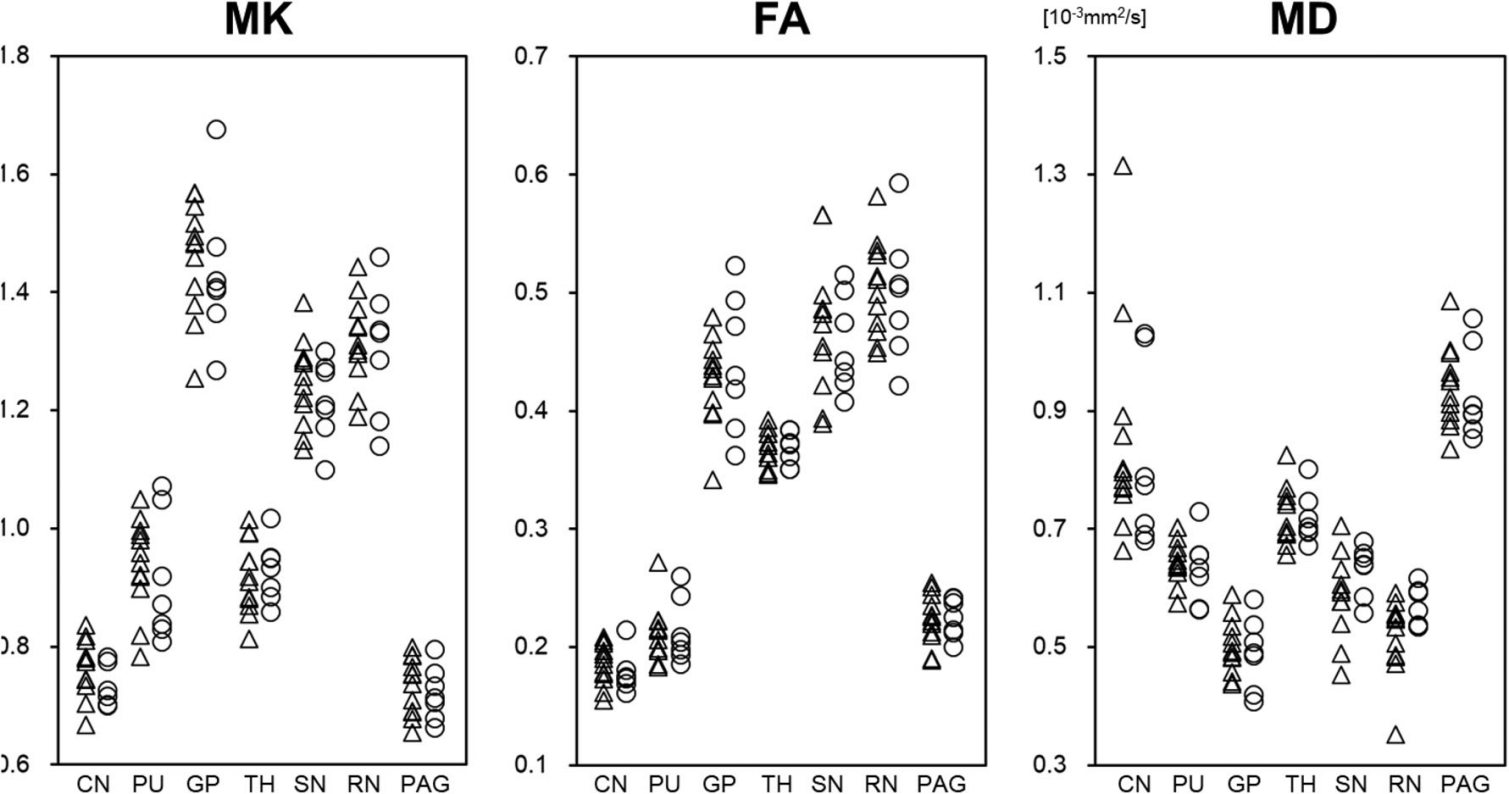
Diffusion kurtosis and tensor metrics of the deep gray matter structures in patients with FGIDs. There were no significant differences in the MK, FA, and MD values in the caudate nucleus (CN), putamen (PU), globus pallidus (GP), thalamus (TH), substantia nigra (SN), red nucleus (RN), and periaqueductal gray matter (PAG) between the groups (Mann–Whitney *U* test). Both the MK and FA values are unitless. (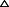) FGIDs, (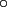) control.

The results of the voxelwise group analysis using TBSS are shown in Figure 1. Compared with healthy controls, MD values in FGID patients were significantly lower in widespread regions of the WM, including the corpus callosum, frontal cortex, posterior limb of the right internal capsule, and the right parahippocampal cingulum. In contrast, no significant differences in the MK or FA values were observed between the two groups. The ROI analysis revealed no significant differences in MK, FA, and MD values in the CN, PU, GP, TH, SN, RN, or PAG between patients with FGIDs and healthy controls (Fig. 2).

The SF‐8 (MCS) demonstrated a significant negative correlation with FA values in the GP (*r* = −0.797, *P* = 0.01 uncorrected) (Table 2), but this effect did not survive after correcting for multiple comparisons. There were no significant correlations between the SF‐8 (PCS) or the five subscale scores of the GSRS and the MK, FA, or MD values in FGIDs patients.

**Table 2:** Correlation between diffusion kurtosis/tensor metrics and SF‐8 (MCS) in FGIDs

	MK			FA			MD		
	*r*	*P*_*uncorr*_	*P*_*fdr*_	*r*	*P*_*uncorr*_	*P*_*fdr*_	*r*	*P*_*uncorr*_	*P*_*fdr*_
PAG	−0.149	0.70	0.96	0.482	0.19	0.49	0.344	0.37	0.57
CN	0.091	0.82	0.96	0.061	0.90	0.90	0.338	0.37	0.57
PUT	0.191	0.62	0.96	−0.241	0.53	0.62	0.224	0.56	0.65
GP	−0.502	0.17	0.96	**−0.797**	**0.01**	0.07	0.536	0.14	0.57
TH	0.018	0.96	0.96	−0.241	0.53	0.62	0.423	0.26	0.57
SN	−0.376	0.32	0.96	0.324	0.40	0.62	−0.317	0.41	0.57
RN	−0.131	0.74	0.96	0.461	0.21	0.49	−0.061	0.89	0.89

CN, caudate nucleus; FA, fractional anisotropy; GP, globus pallidus; MD, mean diffusivity; MK, mean kurtosis; *P*_*fdr*_, *P* value corrected for multiple comparisons using false‐discovery rate; *P*_*uncorr*_, *P* values are uncorrected for multiple comparisons; PAG, periaqueductal gray matter; PU, putamen; RN, red nucleus; SN, substantia nigra; TH, thalamus.

## Discussion

In this study, the MD values in the WM of patients with FGIDs were decreased significantly in widespread regions compared with the healthy controls. Alterations of the FA values in WM regions associated with nociception such as the fornix and insular were previously indicated,^17^ including FA reduction in the splenium of the corpus callosum, right retrolenticular area of the internal capsule, and the right superior corona radiata in IBS.^18^ In addition, using TBSS analysis, the genu of the corpus callosum was found to be negatively correlated with abdominal pain/discomfort intensity in patients with functional constipation and constipation‐predominant IBS (IBS‐C).^19^ Microstructural WM alterations in the right dorsal cingulum bundle in adolescents with IBS may reflect a premorbid brain state or the emergence of a disease‐driven process that results from complex changes in pain‐ and affect‐related processing via spinothalamic and corticolimbic pathways.^20^ Lower FA values in the basal ganglia and higher FA values in the frontal lobe and corpus callosum, as well as a reduced MD in the GP and a higher MD in the thalamus, internal capsule, and coronal radiata have been shown to be associated with abnormalities in the FA of WM connections innervating the viscerotrophic areas of the sensory cortex.^2^, ^21^ Axonal injury may alter synchronization, conduction velocity, and transmission efficiency of neural signals and affect the processing of gastrointestinal pain sensory input or give rise to pain‐related behaviors, including decreased bowel activity.^18^, ^22^, ^23^ Interestingly, we found that FGIDs patients had decreased MD values in widespread WM regions; however, we did not find any significant differences in the MK or FA values. Changes in MD without significant MK and FA changes are rarely observed in neurological disorders. Among the DKI/DTI metrics, an MD decrease is thought to be associated with reduced extracellular water content. ^24^ The MK parameter is said to reflect subtle pathological changes, including increased microgliosis and reactive astrogliosis, or decreased myelin, axonal or neuronal density, ^25^, ^26^, ^27^ whereas FA is believed to reflect more obvious destruction of tissue architecture, such as axonal degeneration, demyelination, and neuronal loss.^28^ Thus, the microstructural changes found in the WM of patients with FGIDs might represent alterations in the volume of extracellular fluid.

We also observed a statistically significant correlation between the SF‐8 (MCS) and the FA values in the GP in FGIDs patients, suggesting that the FA values in the GP could be related to the degree of psychological symptoms with FGIDs. However, we did not find any correlation between the five subscale scores of the GSRS and DKI/DTI measures. The GP is a main component of the basal nuclei, which influence not only exercise function, but also emotion. Compared with the HRQOL, a major advantage is that DKI/DTI can detect subtle alterations in brain microstructure. Therefore, DKI/DTI might be useful to evaluate the degree of SF‐8 (MCS) in FGIDs and the effect of treatments on FGIDs.

IBS studies have reported increased gray matter density (GMD) in the hypothalamus and increased cortical thickness in the anterior midcingulate cortex (aMCC). Correlations between the pain catastrophizing scale and dorsolateral prefrontal cortex (DLPFC) thickness, as well as between pain duration and anterior insula thickness, have been observed among patients with IBS.^29^ Increased GMD in the pregenual anterior cingulate cortex (pgACC) and orbitofrontal cortex (OFC) and decreased GMD in the medial prefrontal cortex (mPFC), DLPFC and posterior cingulate cortex (PCC), ventral striatum, and thalamus have also been reported. ^30^ In female patients with IBS, an increased cortical thickness in the pre‐ and post‐central gyrus as well as decreased thickness in the bilateral insula and left subgenual ACC (sgACC) have been observed.^31^ Smaller gray matter volumes (GMVs) in the insula, cingulate, amygdala, hippocampus, putamen, and frontal regions and a larger GMV in the left postcentral gyrus have also been seen.^32^ Increased thickness in the right posterior insula was associated with longer disease duration in IBS.^33^ The ACC, PFC, insula, and thalamus have been associated with sensory pain in FGIDs with rectal distension.^34^, ^35^ In addition, rectal sensitivity was associated with GMV in the thalamus, insula, PCC, ventrolateral prefrontal cortex (VLPFC), OFC, amygdala, and basal ganglia and negatively associated with GMV in the right thalamus in IBS.^36^ In contrast, in FD, decreased GMD was observed in the bilateral precentral gyrus, mPFC, ACC, MCC, left OFC, and right insula.^37^ Higher GMV in the bilateral putamen and right caudate, and FD‐related differences were primarily located in the amygdala, hippocampus/parahippocampus, thalamus, lingual gyrus, and cerebellum.^38^ Decreased GMD in the right posterior insula, right inferior frontal cortex, and left MCC has also been reported.^39^ Increased FA accompanied by a reduction in mean and radial diffusivity in multiple WM tracts in FD has been reported.^3^

Previous studies have reported IBS and FD separately. However, brain–gut interactions could be related to pathophysiology in FGIDs, including IBS and FD, and the psychological stress could be one cause of visceral pain, which occurs with FGIDs symptoms in both FD and IBS.^40^ However, the numbers were also small in IBS and FD in this pilot study. Thus, in this study, we assessed these as a single FGIDs category. Although remarkable differences in MD values were observed between FGIDs patients and healthy controls, the present study included a small number of FGIDs patients because obtaining informed consent to undergo brain MRI from patients with abdominal symptoms was challenging. In considering the clinical application of our findings, the correlation between severity of FGIDs or HRQOL and DKI/DTI requires cautious interpretation. To confirm our findings, further research is needed in a greater number of patients with FGIDs, including FD and IBS.

## Conclusions

DKI/DTI can help identify the microstructural WM alterations in patients with FGIDs. The FA values in the GP may be useful for a severity assessment of FGIDs.

## Availability of data and materials

The datasets used and analyzed in the current study are available from the corresponding author on demand.

## Ethics approval and consent to participate

The study proposal was reviewed and approved by the Human Ethics Review Committee of Iwate Medical University. Written informed consent was obtained from each patient prior to enrollment.
